# COX-2 Inhibition by Diclofenac Is Associated With Decreased Apoptosis and Lesion Area After Experimental Focal Penetrating Traumatic Brain Injury in Rats

**DOI:** 10.3389/fneur.2019.00811

**Published:** 2019-07-30

**Authors:** Kayvan Dehlaghi Jadid, Johan Davidsson, Erik Lidin, Anders Hånell, Maria Angéria, Tiit Mathiesen, Mårten Risling, Mattias Günther

**Affiliations:** ^1^Experimental Traumatology Unit, Department of Neuroscience, Karolinska Institutet, Solna, Sweden; ^2^Division of Vehicle Safety, Department of Mechanics and Maritime Sciences, Chalmers University of Technology, Gothenburg, Sweden; ^3^Department of Clinical Medicine, Rigshospitalet, University of Copenhagen, Copenhagen, Denmark; ^4^Department of Clinical Neuroscience, Karolinska Institutet, Solna, Sweden

**Keywords:** cyklooxygenase-2, diclofenac, focal penetrating TBI, NSAID, traumatic brain injury

## Abstract

Traumatic brain injury (TBI) is followed by a secondary inflammation in the brain. The inflammatory response includes prostanoid synthesis by the inducible enzyme cyclooxygenase-2 (COX-2). Inhibition of COX-2 is associated with improved functional outcome in experimental TBI models, although central nervous system-specific effects are not fully understood. Animal studies report better outcomes in females than males. The exact mechanisms for this gender dichotomy remain unknown. In an initial study we reported increased COX-2 expression in male rats, compared to female, following experimental TBI. It is possible that COX-2 induction is directly associated with increased cell death after TBI. Therefore, we designed a sequential study to investigate the blocking of COX-2 specifically, using the established COX-2 inhibitor diclofenac. Male Sprague-Dawley rats weighing between 250 and 350 g were exposed to focal penetrating TBI and randomly selected for diclofenac treatment (5 μg intralesionally, immediately following TBI) (*n* = 8), controls (*n* = 8), sham operation (*n* = 8), and normal (no manipulation) (*n* = 4). After 24 h, brains were removed, fresh frozen, cut into 14 μm coronal sections and subjected to COX-2 immunofluorescence, Fluoro Jade, TUNEL, and lesion area analyses. Diclofenac treatment decreased TUNEL staining indicative of apoptosis with a mean change of 54% (*p* < 0.05) and lesion area with a mean change of 55% (*p* < 0.005). Neuronal degeneration measured by Fluoro Jade and COX-2 protein expression levels were not affected. In conclusion, COX-2 inhibition by diclofenac was associated with decreased apoptosis and lesion area after focal penetrating TBI and may be of interest for further studies of clinical applications.

## Introduction

Traumatic brain injury (TBI) is a leading cause of mortality and morbidity worldwide (1). The primary trauma is followed by a secondary inflammatory process that may extend for weeks, involving a multitude of inflammatory mediators (2, 3). Cyclooxygenases (COX) are enzymes that form pro-inflammatory prostanoids from arachidonic acid (4). COX-1 is the constitute isoform while COX-2 is induced by growth factors, cytokines, and inflammatory mediators (5). TBI leads to immediate induction of COX-2 in rat brains, persisting for more than 12 days. COX-2 activity reduces prostaglandin, prostacyclin, and thromboxane products in the brain (6, 7). While COX-2 inhibitors improve cognitive functions and motor performance, the exact function of COX-2 in posttraumatic neuroinflammation is unclear (8–10). Human studies report worse outcomes in women than men after TBI, whereas animal studies report better outcomes in females than males (11). The mechanisms for a possible gender dichotomy remain unknown. In an initial study we reported increased COX-2 expression in male rats compared to female after experimental TBI. The increased COX-2 expression correlated with increased apoptotic cell death detected by TUNEL staining at 24 h, while neuronal necrosis was not observed (12). Due to the possibility of COX-2 induction being directly associated with increased apoptosis after TBI a sequential study to investigate blocking of COX-2 specifically was designed, using the established COX-2 inhibitor diclofenac, in an identical model of focal penetrating TBI (13). The penetrating trauma also allowed for direct administration of diclofenac within the perilesional area. Markers of neuronal degeneration and apoptosis at 24 h after injury, corresponding to the peak of post traumatic inflammation in the brain, were analyzed (14, 15).

## Materials and Methods

The study was conducted in accordance with the Swedish regional Ethics Approval Board for Animal Research (N81/13). A total of 28 male Sprague-Dawley rats (Harlan UK LTD) weighing between 250 and 350 g were divided into the following four groups: experimental trauma + diclofenac treatment (treatment; *n* = 8), experimental trauma and NaCl (controls; *n* = 8), anesthesia and surgery without trauma (sham; *n* = 8), and naïve animals without any manipulation (normal; *n* = 4). The rats were exposed to penetrating focal brain injury as described by Plantman et al. (13) and sacrificed after 24 h. Briefly, the rats were anesthetized by 4% isoflurane and buprenorphine 50 μg/kg. In each rat, a midline incision was made through the skin and periosteum, and a burr hole 2.7 mm in diameter was drilled with its center 3 mm lateral and 3 mm posterior to bregma. Each rat in the experimental trauma and sham groups was placed in a stereotactic frame and positioned with the probe directly above the dura mater. A lead pellet was accelerated by air pressure, hitting a metal cylinder probe with an attached carbon fiber pin, with a tip radius of 1 mm. Depth of penetration into the brain by the pin was limited to 5 mm. Sham operated animals were subjected to identical treatment except for the penetrating injury. Diclofenac (2-(2,6-dichloranilino) phenylacetic acid), 5 μg in 5 μl 0.9% NaCl, was administered immediately following the trauma in the injury cavity in treatment group, and 5 μl 0.9% NaCl was administered in the injury cavity in the “control” group. In the “treatment” and “control” groups, a 23-gauge needle with a 1 mL plastic syringe (Becton, Dickinson and Company) was inserted 4 mm into the cavity and the injection was performed manually with gentle pressure while the needle was carefully withdrawn from the cavity. The total volume was injected without signs of leakage outside the burr hole in any rat. The timing of the injection, equipment used, injection speed, position of the tip of the syringe and total volumes were identical in all animals receiving injections. The dosage was based on previous reports of rodent brain administration of diclofenac (16, 17). The skin lesion was sutured after the drug administration.

**Figure 1:**
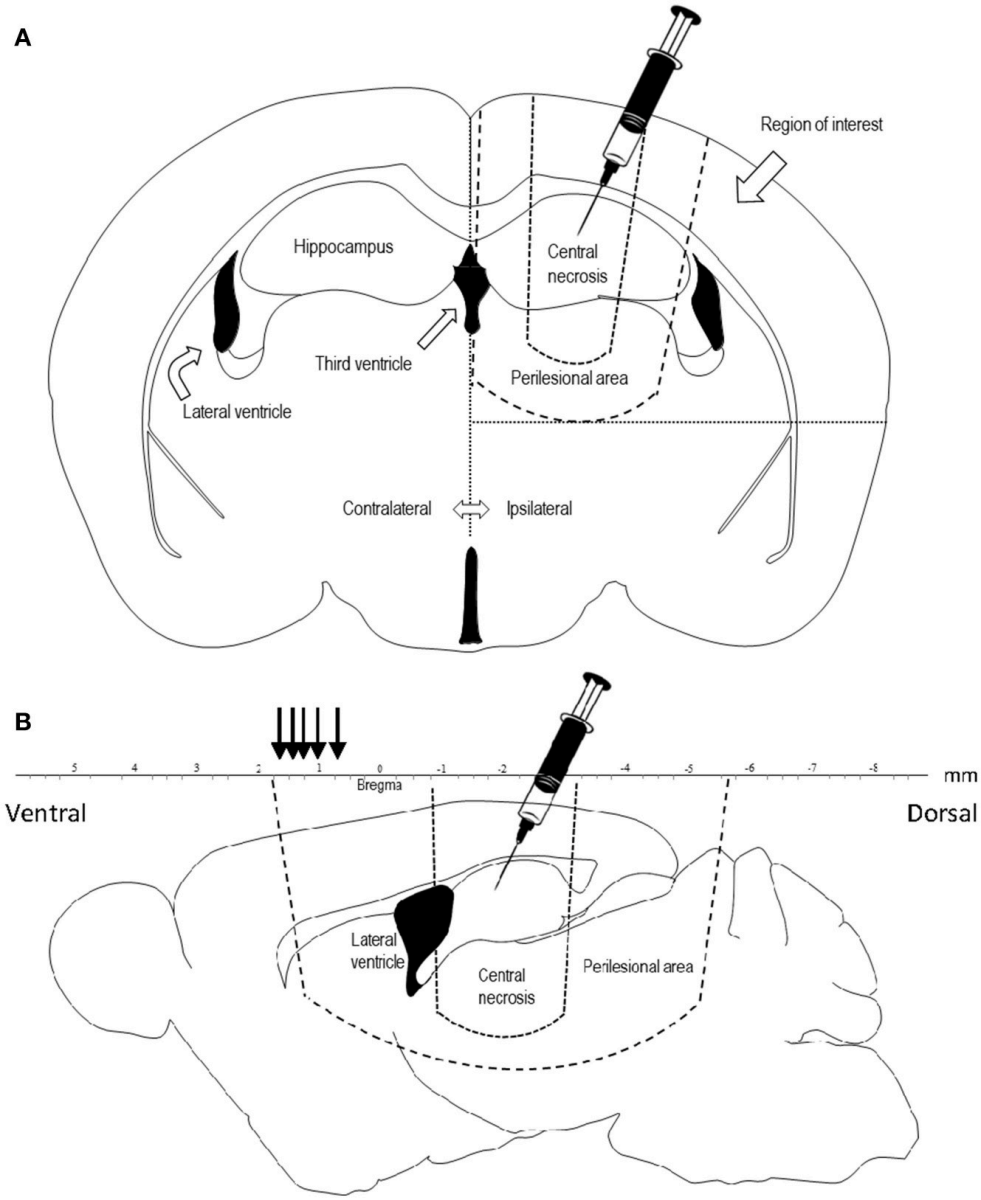
**(A)** Coronal projection of a rat brain, showing the area of penetrating injury and the region of interest for quantification of COX-2, TUNEL, and Fluoro Jade. Diclofenac was injected in the trauma cavity directly after injury. **(B)** Sagittal projection 1.44 mm laterally of the midline of a rat brain showing the central necrosis and perilesional area. Lesion area was measured at comparable sections at bregma +0.48, +0.7, +1.0, +1.2, +1.6, +1.7 mm (black arrows) representing the frontal part of the perilesional area.

The rats were sacrificed by an overdose of pentobarbital, brains were fresh frozen and cut into 14-μm coronal sections using a Microm HM560 cryostat. Each section was cut starting from the level of the trauma, at the level of corpus callosum being visible, approximately at bregma −2.3 mm, and mounted onto Thermo Scientific Superfrost plus slides and stored at −70°C. The region of interest (ROI) was defined medially by the interhemispheric fissure and the midline, basally by the perilesional area and laterally by the lateral border of the right hemisphere (Figures 1A,B). The central necrotic part of the contusion was omitted from the ROI. The slides were blinded to the assessor in all quantifications.

For evaluation of lesion volume, slides from each rat in treatment and control groups were obtained from bregma +0.48, bregma +0.7, bregma +1.0, bregma +1.2, bregma +1.6, and bregma +1.7 for comparison. The lesion areas were calculated at each level in every rat; the assessor was blinded to the treatment group. The lesion areas at each level were compared between the groups and an aggregated mean difference was obtained as a mean difference of the differences at the six different levels. For COX-2 immunofluorescent detection, the slides were rehydrated in phosphate buffer saline (PBS), fixated for 60 min with 4% formalin, and blocked in the solution of 1% bovine serum albumin (BSA), 0.3% Triton X-100 and NaN_3_ (NaAz). The sections were then incubated with the primary antibody (COX-2, dilution 1:100, ab15191 lot: GR146689-1, ABCAM) overnight at 4°C. Next, the slides were incubated with the secondary antibody (Alexa Fluor 488, dilution 1:500, 711-545-152 Jackson ImmunoResearch Laboratories, INC). The nuclear marker used was 4′,6-diamidino-2-phenylindole (DAPI). The TACS 2 TdT- Blue Label *in situ* Apoptosis Detection Kit, by Terminal Deoxynucleotidyl transefarse dUTP nick end labeling (TUNEL), was used for the detection of apoptosis in the tissue sections. The slides were put on a heated plate for 30 min and then fixated in 3.7% formaldehyde. The slides were then permeabilized in ethanol for 5 min in −20°C and put in a quenching solution (94 ml methanol and 6 ml 50% hydrogen peroxide) for 5 min before being treated with TdT labeling, incubated for 60 min at 37°C and stopped with a 1XTdT stop buffer. Before detection, the slides were incubated with Streptavidin-HRP (1:500) for 20 min and followed by counter staining with Nuclear Fast Red for 2 min. The slides were then dehydrated in a series of dH_2_O, 70% ethanol, 90% ethanol, 100% ethanol and xylene, and finally mounted in Entellan. To detect degenerating neurons, the slides were fixated in 4% formalin and immersed in 0.06% potassium permanganate (KMnO_4_) for 10 min before being incubated for 30 min in Fluoro Jade B. The slides were dried at 50°C, immersed in xylene for 15 min and mounted with Entellan.

Sections were first examined at 1x magnification through a Nikon E600 microscope using dark field illumination. The level of each examined coronal section was identified using the 3rd edition of Paxinos and Watson's rat brain atlas (18). The lateral ventricles, anterior commissure and the shape of the corpus callosum were used as landmarks. For COX-2 protein expression detection, eight sections per animal were digitally photographed in 40x using dark field illumination in a Nikon Eclipse E600 microscope. Eight whole brain sections were photographed per animal. In the ipsilateral side one picture was taken from the perilesional area and one from the ventrolateral cortex, with corresponding areas being photographed in the contralateral side. Thus, 32 pictures (four pictures per brain section and eight sections per animal) were analyzed for each animal. The integrated intensity of the COX-2 protein expression was analyzed in ImageJ. The following macro-code was used as the batch process for automated counting of the integrated intensity with a set threshold: run(“8-bit”);run(“Invert”);setAutoThreshhold(“Yen”);//run (“Threshold…”); run(“Measure”);close();saveAs(“Results” …). Quantification of Fluoro Jade and TUNEL positive cells was done manually in 40x magnification. Fluoro Jade positive cells were counted in the ipsilateral and contralateral side of eight sections per animal. TUNEL positive cells were counted similarly in four sections per animal. The lesion area was analyzed by Fiji/ImageJ software from 1x dark field photographs.

### Statistical Analyses

Statistical analyses were carried out in GraphPad Prism version 6.05 for Windows (GraphPad Software, La Jolla, USA). All error bars represent the standard error of the mean (SEM). COX-2, TUNEL and Fluoro Jade was analyzed by the non-parametric Kruskal Wallis ANOVA followed by Benjamini Hochberg *post-hoc* test. Lesion area was analyzed by two-way unpaired *t*-test. *p*<0.05 was considered significant. Significance levels: ^*^p<0.05, ^***^p < 0.005.

## Results

**Figure 2:**
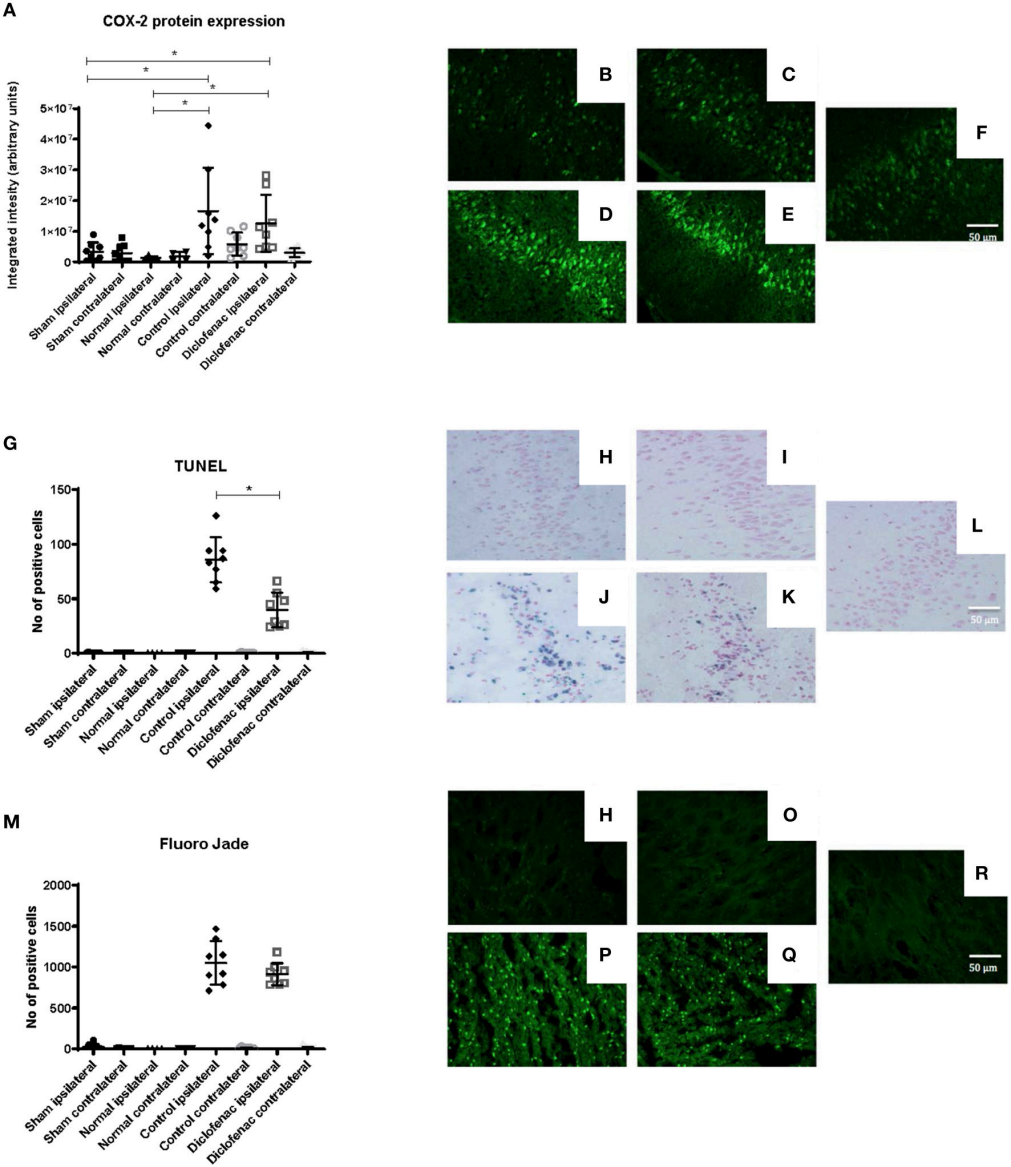
**(A)** COX-2 protein expression was upregulated 24 h following injury. Diclofenac did not affect protein expression. Photomicrographs of COX-2 fluorescence **(B)** “normal” ipsilateral **(C)** “sham” ipsilateral **(D)** “control” trauma ipsilateral **(E)** “diclofenac” treated trauma ipsilateral. **(F)** Contralateral side **(G)** TUNEL staining showing lower apoptosis levels by diclofenac treatment. Photomicrographs of TUNEL **(H)** “normal” ipsilateral **(I)** “sham” ipsilateral **(J)** “control” trauma ipsilateral **(K)** “diclofenac” treated trauma ipsilateral. **(L)** Contralateral side **(M)** diclofenac treatment did not affect Fluoro Jade detected neuronal degeneration. Photomicrographs of Fluoro Jade **(N)** “normal” ipsilateral **(O)** “sham” ipsilateral **(P)** “control” trauma ipsilateral **(Q)** “diclofenac” treated trauma ipsilateral. **(R)** Contralateral side. Data expressed as mean ± SEM. ^*^p < 0.05.

**Figure 3:**
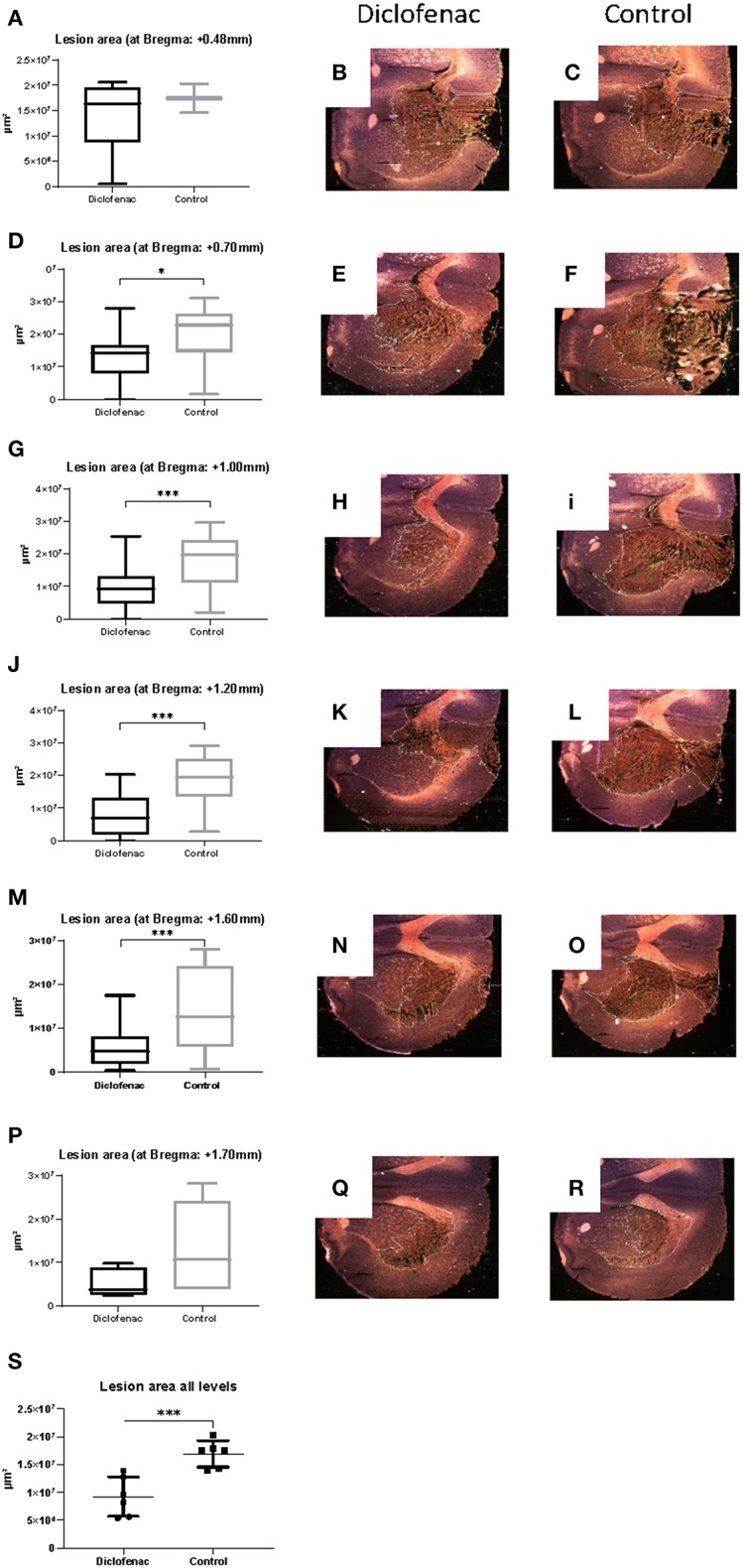
**(A–S)** Diclofenac treatment decreased lesion area in the frontal perilesional area. Lesion area was measured at comparable sections at bregma +0.48, +0.7, +1.0, +1.2, +1.6, +1.7 mm (black arrows) representing the frontal part of the perilesional area. Data expressed as mean ± SEM. ^*^p < 0.05, ^***^p < 0.005.

COX-2 protein expression was increased in the ipsilateral side following trauma, in both the “controls” and “diclofenac treatment” groups compared to the “sham” and “normal” (*p* < 0.05) groups. No differences were observed between the “controls” and the “diclofenac” group. Weak COX-2 expression was seen in the contralateral side in all groups except for “normal” (Figures 2A–F). TUNEL positive cells were increased in the injured hemispheres of the “diclofenac” and “control” groups compared to the “sham” and “normal” groups. TUNEL positive cells were not detected in the contralateral hemispheres. The number of TUNEL positive cells were lower in the diclofenac treatment group compared to the “control” group with a mean change of 54% (“diclofenac” 39.8 cells/view—“control” 85.6 cells/view) (*p* < 0.05) (Figures 2G–L). Fluoro Jade positive cells were increased in both the “diclofenac” and “control” groups compared to “sham” and “normal.” Positive cells were not detected in the contralateral sides. Furthermore, no differences were detected between the different groups (Figures 2M–R). The lesion area was measured in the groups exposed to penetrating trauma. The lesion areas measured at six different levels were significantly smaller at four levels and smaller with statistical significance at four. The mean differences were 55% (“diclofenac” 9.2 mm^2^–“control” 16.9 mm^2^) (*p* < 0.005) (Figures 3A–S).

## Discussion

This study showed that specific COX-2 inhibition by diclofenac was associated with decreased apoptosis in the perilesional area after focal penetrating TBI. The focal penetrating TBI model is highly reproducible and has a known gender specific COX-2 response (12). In this specific context, the model mimics an open brain contusion, which is a particular form of head trauma (19). This kind of injury allowed direct intralesional administration of diclofenac. The mode of administration is unusual but provides direct access to the intended target area and reduces the risk of a heterogenous uptake in the brain from intravenously or intraperitoneally injected substances. Intralesional administration in the brain is not standard clinical practice in head injuries, which may comprise a limitation of the clinical applicability. Direct administration into surgical sites is, however, practiced with gliadel wafers for brain tumor therapy and antibiotics for management of ventriculitis; hence intra-axial administration for clinical focal brain injuries should be considered as a novel clinical option. There are potential risks associated with intralesional administration of a vasoactive substance, and coagulation derangements including a possible higher risk of hemorrhage. Safety needs to be addressed in clinical studies since our study only intended to analyse the impact of COX-2 inhibition on cell death; the large effect size that may foster hopes of neuroprotection was not expected. We targeted the immediate inflammation known to occur 24 h after TBI (14) and found important differences probably caused by diclofenac treatment. Yet, the present study cannot determine the precise effects of diclofenac; a complete time series is necessary to evaluate final cell survival and comprehensive kinetics.

COX-2 was upregulated in the perilesional area after TBI, similarly to our experience (12) and previous reports (8, 10, 20–22). Most expression was found in the ipsilateral hemisphere, with little expression also in the contralateral side. Diclofenac did not decrease protein expression of COX-2. Earlier studies report conflicting results of COX inhibition. The COX-2 inhibitor rofecoxib did not alter the COX-2 expression (21), while the COX-2 inhibitor DFU decreased COX-2 when the drug was administered 10 min before trauma, but not when administered 2–6 h post trauma (10). It is possible that systemic inflammation in the body is sufficient for general COX-2 induction, and that the acute blood brain barrier disruption by the focal injury allowed for COX-2 to enter the brain.

TUNEL staining, indicative of apoptosis was increased in the perilesional area in all trauma groups compared to the “sham” group. Diclofenac decreased TUNEL staining compared to “control.” The number of neurons positive for Fluoro Jade were increased after TBI, although they were not affected by diclofenac. Fluoro Jade labels degeneration, both necrosis and apoptosis, while TUNEL staining is specific in labeling apoptosis (23). Fluoro Jade is specific in labeling neurons, while TUNEL labels all cell types undergoing apoptosis. It is therefore possible that the inflammatory amelioration by diclofenac occurs primarily in cells other than neurons. It is also possible that high levels of neuronal necrosis resulting from the penetrating injury conceals possible anti-apoptotic effects in the neurons specifically. DFU decreased activated caspase-3 levels, further suggesting specific antiapoptotic effects (10), while no effects on TUNEL or Fluoro Jade staining were detected after rofecoxib treatment following rodent fluid percussion TBI (21). Potentially, COX-2 inhibition is more effective in specific TBI subgroups, which should be explored in future studies. The amount of TUNEL positive cells in the “control” group (male rats) corresponded to the amount found in male rat brains when comparing male and female responses in an identical setting in the preceding study (12). TUNEL positive cells in the “diclofenac” group also corresponded to the amount in untreated female rats exposed to identical trauma. It is possible that lower levels of COX-2 in female brains is protective, and that male subgroups should be targeted in future studies of neuroprotective properties of diclofenac. TUNEL positive cells in the “control” (NaCl vehicle) group corresponded to the amount in male rats receiving no injection in the preceding study (12), why possible cell death resulting from the injection only could be excluded.

Diclofenac appeared to decrease the macroscopic lesion area after 24 h. We could only quantify and compare the lesion areas in a limited number of brain sections which were available after the immunohistochemical analyses; the extensive effect on lesion size was unexpected and serendipitous. We did not have material to make a three-dimensional analysis of the lesion volume, which comprised a limitation. The size effect was, however, extensive from the analyses we made and the intralesional treatment needs to be evaluated with prospective quantification of lesion volumes. Similar results of COX-2 inhibition reducing lesion size together with increased glia cell proliferation in the perilesional area have been reported (9). It is therefore possible that the anti-apoptotic and tissue sparing effects were not a direct effect by COX-2 inhibition but mediated by other cells. Microglial activation occurs within 24 h after experimental TBI (24). Sites of activation often coincide with neuronal degeneration and axonal abnormality, hence anti-inflammatory treatments targeting microglia are suggested as potential therapeutic strategies (25). It is also feasible that astrocytes mediate apoptosis. Astrocytes maintain the homeostasis of ions, transmitters, water, and blood flow, critical for neuronal function. In response to TBI, astrocytes become active in response to axonal injury, vascular disruption, ischemia and inflammation (26), and are emerging as both potent pro-inflammatory and anti-inflammatory cells (27). Astrocytes are associated with T-cell apoptosis in autoimmune inflammation (28). Future studies should therefore aim at investigating specific apoptotic pathways and the effect of diclofenac on neurons as well as glia cells. These studies may also target protein markers of axonal functionality, such as phosphorylated neurofilaments to determine if diclofenac in addition to reducing cell death favors the restoration of axonal functionality decreased by injury.

The findings suggest that the gender-related difference in apoptotic cells after TBI (12) is associated with COX-2 regulation. Most anti-inflammatory drugs have failed to produce lasting effects (29). It is possible that COX-2 inhibition is beneficial in focal TBI, making diclofenac a potential candidate for further clinical applications. In addition, local administration of the drug may provide a novel treatment that can be developed clinically.

## Conclusion

COX-2 inhibition by diclofenac is associated with decreased apoptosis after focal penetrating TBI and may be beneficial in preventing brain tissue damage.

## Ethics Statement

Swedish regional ethics approval board for animal research (N81/13).

## Author Contributions

MG, MR, JD, and TM planned the study. MG, JD, and KD performed the experiments. KD, EL, MA, and AH performed preparation and analysis. All authors contributed to the text.

### Conflict of Interest Statement

The authors declare that the research was conducted in the absence of any commercial or financial relationships that could be construed as a potential conflict of interest.
